# Metabolic Profile, Bioavailability and Toxicokinetics of Zearalenone-14-Glucoside in Rats after Oral and Intravenous Administration by Liquid Chromatography High-Resolution Mass Spectrometry and Tandem Mass Spectrometry

**DOI:** 10.3390/ijms20215473

**Published:** 2019-11-03

**Authors:** Feifei Sun, Haiguang Tan, Yanshen Li, Marthe De Boevre, Sarah De Saeger, Jinhui Zhou, Yi Li, Zhenghua Rao, Shupeng Yang, Huiyan Zhang

**Affiliations:** 1 Institute of Animal Sciences, Chinese Academy of Agricultural Sciences, Beijing 100193, China; sunff@ahau.edu.cn (F.S.); raozhenghua@caas.cn (Z.R.); 2 Institute of Apicultural Research, Chinese Academy of Agricultural Sciences, Key Laboratory of Bee Products for Quality and Safety Control, Bee Product Quality Supervision and Testing Center, Ministry of Agriculture, Beijing 100093, China; tanhaiguang1994@foxmail.com (H.T.); zhoujinhui@caas.cn (J.Z.); liiy01@caas.cn (Y.L.); 3 College of Animal Science and Technology, Anhui Agricultural University, Hefei 230036, China; 4 College of Life Science, Yantai University, Yantai 264005, China; liyanshen@tyu.edu.cn; 5 Centre of Excellence in Mycotoxicology and Public Health, Faculty of Pharmaceutical Sciences, Ghent University, Ottergemsesteenweg 460, 9000 Ghent, Belgium; Marthe.DeBoevre@UGent.be (M.D.B.); Sarah.DeSaeger@UGent.be (S.D.S.)

**Keywords:** masked mycotoxins, zearalenone, metabolism, bioavailability, risk assessment

## Abstract

Zearalenone-14-glucoside (ZEN-14G), a key modified mycotoxin, has attracted a great deal of attention due to the possible conversion to its free form of zearalenone (ZEN) exerting toxicity. In this study, the toxicokinetics of ZEN-14G were investigated in rats after oral and intravenous administration. The plasma concentrations of ZEN-14G and its major five metabolites were quantified using a validated liquid chromatography tandem mass spectrometry (LC-MS/MS) method. The data were analyzed via non-compartmental analysis using software WinNonlin 6.3. The results indicated that ZEN-14G was rapidly hydrolyzed into ZEN in vivo. In addition, the major parameters of ZEN-14G following intravenous administration were: area under the plasma concentration–time curve (AUC), 1.80 h·ng/mL; the apparent volume of distribution (V_Z_), 7.25 L/kg; and total body clearance (CL), 5.02 mL/h/kg, respectively. After oral administration, the typical parameters were: AUC, 0.16 h·ng/mL; V_Z_, 6.24 mL/kg; and CL, 4.50 mL/h/kg, respectively. The absolute oral bioavailability of ZEN-14G in rats was about 9%, since low levels of ZEN-14G were detected in plasma, which might be attributed to its extensive metabolism. Therefore, liquid chromatography high-resolution mass spectrometry (LC-HRMS) was adopted to clarify the metabolic profile of ZEN-14G in rats’ plasma. As a result, eight metabolites were identified in which ZEN-14-glucuronic acid (ZEN-14GlcA) had a large yield from the first time-point and continued accumulating after oral administration, indicating that ZEN-14-glucuronic acid could serve a potential biomarker of ZEN-14G. The obtained outcomes would prompt the accurate safety evaluation of ZEN-14G.

## 1. Introduction

Zearalenone (ZEN), produced by the filamentous *Fusarium* fungi, is one of the most frequently occurring *Fusarium* mycotoxins [1,2]. ZEN can react with estrogenic receptors, exhibiting estrogen-like activity [3]. The modified forms of mycotoxins have recently attracted considerable attention [4,5]. Food and feed contaminated with modified mycotoxins such as ZEN-14-glucoside (ZEN-14G) or deoxynivalenol-3-β-D-glucoside (DON-3G) could result in potential underestimation of the exposure [4,6]. Thus, the European Commission stated that it is appropriate to evaluate the exposure to modified forms of various toxins in addition to the parent compounds [7], since the modified forms might be hydrolyzed into the parent toxins, exerting toxicity [8,9]. Furthermore, the estrogenic potency of ZEN-14G is comparable to that of ZEN [7].

Over recent years, a great deal of research has been done concerning the metabolism, contribution, bioavailability, hydrolysis, and toxicokinetic characterization of DON and DON-3G [10,11,12,13,14], which consequently laid the foundation for the establishment of a tolerable daily intake of DON. It was reported that after oral administration of DON-3G, only DON was detected in plasma, and the absolute oral bioavailability of DON-3G in broiler chickens and pigs was 3.79% and 16.1%, respectively [13]. Meanwhile, the relevant studies on ZEN toxicokinetics and metabolism have also been extensively investigated [15,16,17,18,19]. The oral bioavailability of ZEN was very low (10% in poultry and rats), and extensive metabolism of ZEN was observed [20,21,22,23]. Additionally, ZEN-14G was reported to be hydrolyzed via intestinal microbes [24]. Our research group evaluated the metabolism of ZEN-14G in vivo and in vitro. Consequently, we found that ZEN-14G could undergo deglycosylation not only via intestinal microbes, but also in the liver. The metabolic pathways of ZEN-14G were described in detail [25]. However, the knowledge on its toxicokinetic behavior is still scarce to absent. The paucity of toxicokinetic information hinders the authentic estimation of dietary exposure of livestock and humans to ZEN and its modified forms. To accurately assess the exposure to mycotoxins, it is of great importance to carry out toxicokinetic studies of ZEN-14G.

## 2. Results and Discussion

### 2.1. Metabolic Profile of ZEN-14G in Rat Plasma Using Liquid Chromatography High-Resolution Mass Spectrometry (LC-HRMS)

High-resolution mass spectrometry (HRMS) and triple quadrupole coupled to mass spectrometry (MS/MS) have been widely used in various fields, including metabolism, environmental toxicity, and safety evaluation. Both techniques can be used for qualification and quantification. In general, MS/MS can acquire better sensitivity when performed in multiple reaction monitoring mode. HRMS has a far greater advantage in confirmatory capabilities over MS/MS instruments. Therefore, HRMS was utilized for investigation of the metabolites’ identification and elucidation.

**Figure 1:**
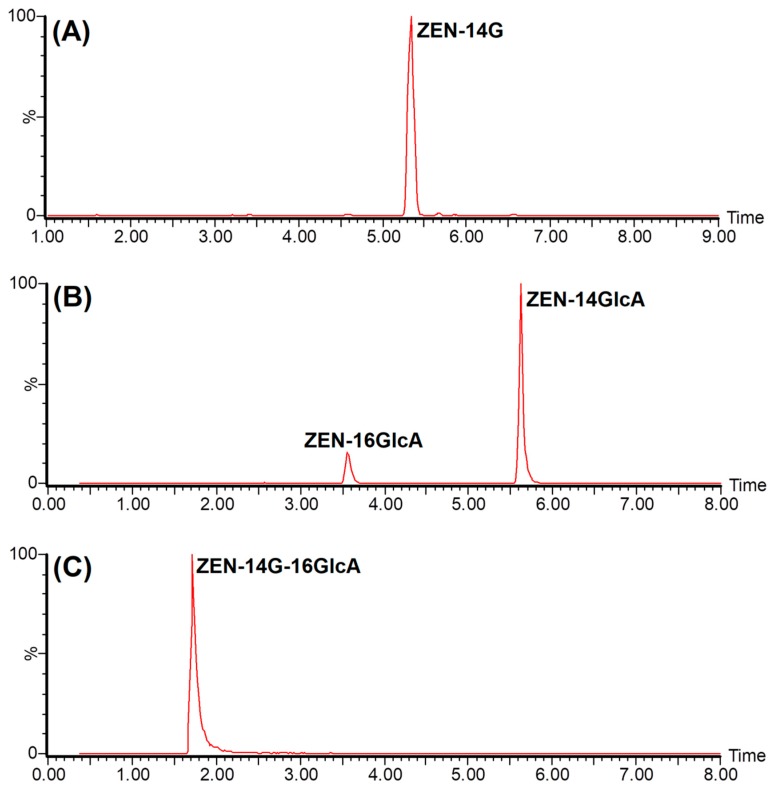
Representative extracted ion chromatograms (EICs, the extraction window was 50 mDa) of ZEN-14-glucoside (ZEN-14G, *m/z* 479.19227) (**A**) and its major phase II metabolites ZEN-14-glucuronic acid (ZEN-14GlcA, *m/z* 495.18719) (**B**) and ZEN-14G-16GlcA (*m/z* 655.22436) (**C**) detected in rat plasma after intravenous injection (i.v.) and oral administration (p.o.).

It has been reported that glucuronidation is the predominant pathway for ZEN-14G while ZEN-14-glucuronic acid (ZEN-14GlcA) and ZEN-14G-16GlcA are the major phase II metabolites of ZEN-14G in rats [25]. Thus, the authors assumed that ZEN-14G was rapidly transformed into other conjugates in plasma. Because the reference standards of ZEN-14GlcA and ZEN-14G-16GlcA are not commercially available, the relative quantification of the conjugates was based on the peak area of each compound. Therefore, the plasma samples were re-determined using a semi-quantitation method via LC-HRMS, which had been successfully applied to investigate the in vitro metabolism of ZEN-14G and ZEN in livestock and humans [19,25]. Consequently, a large number of glucuronic acid conjugates were discovered in plasma, and ZEN-14GlcA and ZEN-14G-16GlcA were predominant. The representative extracted ion chromatograms (EICs) for ZEN-14G, ZEN-14GlcA, and ZEN-14G-16GlcA from plasma samples are illustrated in Figure 1.

**Figure 2:**
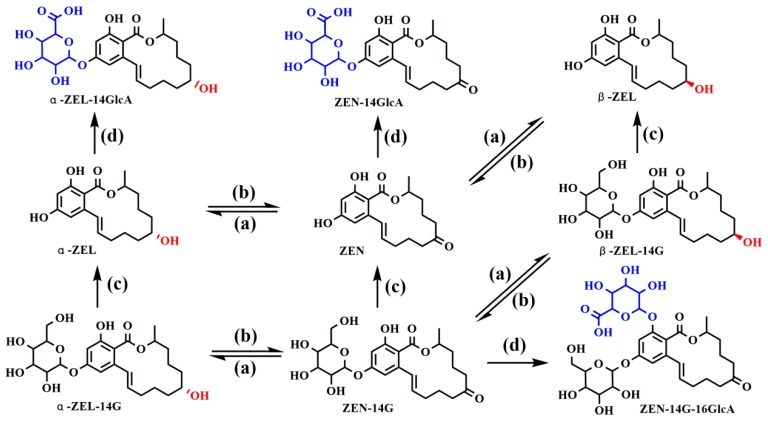
The chemical structures of ZEN-14G metabolites observed in plasma, and their proposed metabolic pathways after oral and intravenous injection (i.v.) administration, including (**a**) hydrogenation, (**b**) dehydrogenation, (**c**) deglycosylation, and (**d**) glucuronidation.

To have an integrative characterization of ZEN-14G in plasma, the proposed metabolic pathways are illustrated in Figure 2. Hydrogenation, glucuronidation, hydrolysis, and hydrolysis-based glucuronidation were the metabolic routes for ZEN-14G in rats’ plasma. After both oral and intravenous administration, a substantial amount of ZEN-14GlcA was detected, implying hydrolysis-based glucuronidation at the C-14 position. Note that the hazard of ZEN-14GlcA is solely due to its hydrolysis to the aglycone after digestion in mammals or cleavage via human microbiota [26,27]. Therefore, the toxicity of ZEN-14GlcA is definitely not negligible.

### 2.2. Method Validation

Considering the matrix influence, matrix-matched calibration curves were used. The limits of quantitation (LOQs) for ZEN-14G, α-ZEL-14G, β-ZEL-14G, α-ZEL, β-ZEL, and ZEN were 0.03, 0.03, 0.1, 0.1, 0.1, and 0.1 ng/mL, respectively. An acceptable linearity for each compound was observed in the observed range with the coefficient of correlation over 0.99. Precision and accuracy were evaluated by spiking at three different concentration levels (0.03, 0.15, 0.30 ng/mL for ZEN-14G and α-ZEL-14G, and 0.10, 0.50, 1.00 for β-ZEL-14G, α-ZEL, β-ZEL, and ZEN). The mean recoveries for six compounds at three concentrations ranged from 79.2% to 92.4%. The intra-day and inter-day precision at three concentrations were all below 15%. The results indicated that the validated method met the requirements, and could be used for clinical practice. The method validation information and mass spectrometric parameters of these six compounds are attached in Tables S1 and S2.

### 2.3. Toxicokinetic Characterization

The validated LC-MS/MS method was eligible for the toxicokinetic characterization of ZEN-14G in rats. The obtained data (above the LOQ) were analyzed via non-compartmental analysis using WinNonlin 6.3 software (Pharsight Co. Mountain View, CA, USA). The area under the plasma concentration–time curve (AUC) was obtained using the trapezoidal rule with extrapolation to infinity. The main parameters of ZEN-14G following intravenous administration were: the elimination half-life (Elimination t_1/2_), 0.22 h; the area under the concentration–time curve from 0 to the ultimate blood collection point (AUC_last_), 1.79 h·ng/mL; the area under the concentration–time curve from 0 to the last blood collection time point (AUC_0-infinity_), 1.80 h·ng /mL; the apparent volume of distribution (V_Z_), 87,247.73 mL/kg; the apparent body clearance (CL), 277,502.12 mL/h/kg; and the mean residence time (MRT), 0.20 h, respectively (Table 1). After oral administration, the plasma concentrations of ZEN-14G were detected at low levels, and the major parameters were as follows: Elimination t_1/2_, 0.96 h; AUC_last_, 0.11 h·ng/mL; AUC_0-infinity_, 0.16 h·ng/mL; V_Z_, 6.24 mL/kg; CL, 4.5 mL/h/kg; and MRT, 0.60 h, respectively (Table 1). The half-life period of ZEN in pigs was much longer than ZEN-14G in rats [17], which may be attributed to enterohepatic circulation. It has been reported that the absolute oral bioavailability of ZEN is around 51.5% in female pigs [17], but for chickens (broilers, laying hens, or turkey poults), the absolute bioavailability is between 6.8%–10.3% [20]. However, the absolute oral bioavailability of ZEN-14G was 8.89%. The low absolute bioavailability of ZEN-14G might be due to the deglycosylation of ZEN-14G to ZEN. The CL and V_Z_ are extremely different between oral (p.o.) and intravenous (i.v.) administration. The V_Z_ depends on the total mass absorbed and the plasma concentration. For oral administration, the low plasma concentration of ZEN-14G may because ZEN-14G appeared to be distributed into a very large volume or rapidly biotransformed into other compounds. The low oral bioavailability and low plasma concentration of ZEN-14G led to the lower V_Z_ than that of i.v. administration. Relative to ZEN-14G, ZEN was found to have a large volume of distribution, implying that ZEN is more likely to be distributed to tissues [17]. CL is the volume of blood from which a compound is removed per unit time. In comparison with p.o., much more of ZEN-14G was absorbed in the plasma after intravenous injection (i.v.)—more volume of blood cleared of ZEN-14G per unit time.

**Figure 3:**
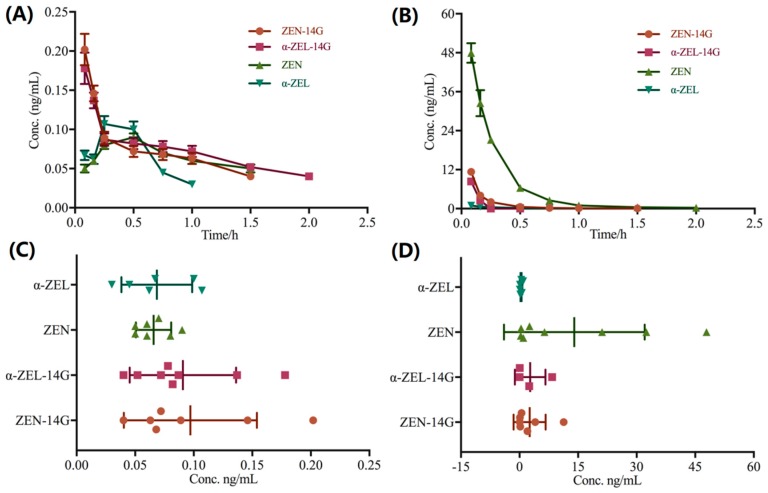
(**A**) Plotted mean plasma concentration versus time curves of ZEN-14G and its quantifiable metabolites (α-ZEL-14G, α-ZEL, and ZEN) in Wistar rats after oral administration. α-ZEL-14G and ZEN-14G were the principle metabolites in plasma with the same elimination tendency. (**B**) Plotted mean plasma concentration versus time curves of ZEN-14G and its quantifiable metabolites after intravenous injection. ZEN-14G was rapidly converted into ZEN. (**C**) General distribution of ZEN-14G and its quantifiable metabolites in Wistar rats’ plasma after oral administration (the same data set as panel A). ZEN-14G and its quantifiable metabolites were detected at very low levels. (**D**) The distribution of ZEN-14G and its quantifiable metabolites in Wistar rats’ plasma after intravenous administration (the same data set as panel B). ZEN was the predominant derivative in plasma following intravenous injection.

After oral administration, the peak concentration of ZEN-14G was only 0.2 ng/mL at the first-time point (5 min), and sharply dropped down. α-ZEL-14G, the hydrogenated product of ZEN-14G, was detected as well, and had the same tendency as ZEN-14G. Observed in Figure 3A, α-ZEL and ZEN were also detected in plasma after oral administration, but only at a trace level. In Figure 3C, it was obvious that ZEN-14G and its hydrogenated derivate were the major metabolites, however in a very low amount. The rapid and extensive metabolism of ZEN-14G would account for the low concentrations of the quantitated metabolites. In Figure 3B, after intravenous administration at the same dosage, a relatively larger amount of ZEN-14G was detected in comparison with oral administration. However, the yield of ZEN—the deglycosylated metabolite of ZEN-14G—was approximately 4.5 times higher than that of ZEN-14G, suggesting that ZEN-14G had the propensity to undergo deglycosylation to form ZEN, which was different from oral administration. It is clear from Figure 3D that a small amount of α-ZEL and α-ZEL-14G was observed in plasma as well.

**Figure 4:**
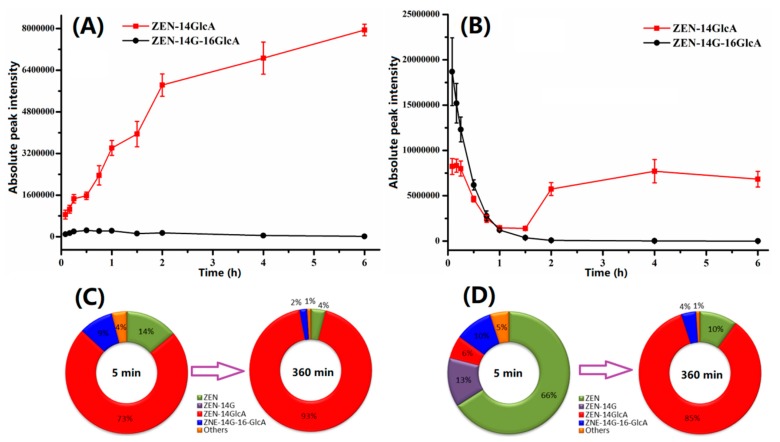
(**A**) Profile of ZEN-14GlcA and ZEN-14G-16GlcA after oral administration of ZEN-14G. ZEN-14GlcA continued accumulating since the first time-point. (**B**) Profile of ZEN-14GlcA and ZEN-14G-16GlcA after intravenous injection of ZEN-14G. The fluctuation of ZEN-14GlcA was observed. (**C**) Dynamic profile of different components in plasma after oral administration. ZEN-14GlcA was dominant throughout the whole study. (**D**) Dynamic profile of different components in plasma following intravenous administration. ZEN-14G was swiftly deglycosylated into ZEN after intravenous injection and then bound with glucuronic acid, generating ZEN-14GlcA.

It is important to highlight that on the basis of relative quantitation of the principal conjugates, the conjugate of ZEN-14G at the C-16 position was determined, however at a strikingly low level, whereas ZEN-14GlcA was detected at the first time-point, and the amount of ZEN-GlcA kept growing until the last time point (Figure 4A). Figure 4C shows the dynamic profile of different components in plasma after oral administration. ZEN-14GlcA was dominant throughout the whole study, implying that ZEN-14G tended to undergo deglycosylation mediated by the carboxylesterases in plasma by generating ZEN. Meanwhile ZEN had the tendency to trigger conjugation, producing ZEN-14G, which was in agreement with ZEN [9,28]. However, the accumulation of ZEN-14GlcA might have the potential risk to release ZEN via intestinal microbes [24]. The double-peak phenomenon of ZEN observed in pigs confirmed the possibility of converting ZEN-14GlcA to ZEN [29,30]. Thus, though the oral bioavailability of ZEN-14G was extremely low (8.89%), the risk of exposure to ZEN-14G still remained. Additionally, ZEN-14GlcA has been reported to be the primary derivative of ZEN [19]. Therefore, the high yield and slow elimination of ZEN-14GlcA make it a potential biomarker for monitoring ZEN and its modified form, ZEN-14GlcA. Besides that, the major toxicokinetic parameters of ZEN-14G following oral administration are shown in Table 1. The half-life, area under the concentration vs. time curve, total body clearance, and mean residence time were 0.96 h, 0.16 h/ng/mL, 4.5 mL/h/kg, and 1.36 h, respectively, indicating that ZEN-14G was extraordinarily metabolized and rapidly eliminated after oral administration. It has been reported that ZEN is characterized by a low oral bioavailability and rapid elimination in poultry, where conjugation is the main reaction [23]. However, a bioavailability of approximately 80%–85% has been estimated following a single oral dose of 10 mg/kg b.w. ZEN, in that reduced ZEN metabolites may easily cross biological membranes and enterohepatic circulation was observed in pigs [29]. However, the oral bioavailability of ZEN-14G was much lower than that of ZEN. This might be attributed to the introduction of glycoside at the C-14 position, increasing the polarity and reducing the permeability of ZEN-14G. In Figure 4B, the amount of ZEN-14G-16GlcA decreased as time went by, whereas ZEN-14GlcA dropped before 1.5 h post administration, increased afterwards, and remained stable. This phenomenon indicated that the glucuronic acid conjugate of ZEN-14G at the C-16 accounted for the majority of the derivatives. This conjugate might be degraded into ZEN and then generate ZEN-14GlcA, which is consistent with reported results that the major conjugate of ZEN was ZEN-14GlcA [19]. Besides, the fluctuation of ZEN-14G was likely to be caused by the enterohepatic circulation of ZEN, and enterohepatic circulation was observed previously [17,29,30]. Figure 4D depicts the dynamic distribution of the parent toxin and its derivatives at the typical time points before and after the peak of ZEN (the first and last time points).

## 3. Materials and Methods

### 3.1. Chemicals and Reagents

The preparation of ZEN-14G, α-ZEL-14G, and β-ZEL-14G was described in our previous published paper [25]. Zearalenone, α-zearalenol (α-ZEL), and β-zearalenol (β-ZEL) were commercially available from Fermentek Ltd (Jerusalem, Israel). Acetonitrile and formic acid were LC-MS grade and purchased from ThermoFisher Chemical Co. (Harnover Park, IL). Water was purified from a Milli-Q system (Bedford, MA, USA). Other chemicals and reagents used in this study were of analytical grade.

### 3.2. Animals and Experimental Protocol

ZEN-14G was dissolved in aqueous solution containing 0.5% carboxymethyl cellulose. We selected twelve Wistar rats (half male and half female, with an average weight of 180 g, obtained from Vital River Laboratory Animal Technology Co. Ltd (Beijing, China). The rats were raised for one week for acclimation before the experiment, and the rats went through a 12 h fast before dosing. Six rats were treated with ZEN-14G via oral gavage at a dosage of 0.75 mg/kg body weight followed by blood collection at 0.083, 0.16, 0.25, 0.5, 0.75, 1.0, 1.5, 2, 4, and 6 h post administration while the other six rats were administered with the same amount of ZEN-14G intravenously and the blood samples were collected at the same time points. After a one-week withdrawal period, a crossover design was used for the following experiment where the rats administered orally would receive intravenous administration and vice versa. No visible adverse phenomena were observed after any route of administration. All the animal experiments complied with the National Institutes of Health guide for the care and use of laboratory animals, which has been approved on 20 March 2018 by the ethical committee of China Agricultural University (Beijing, China) (2018-SYXK-0238).

### 3.3. Plasma Sample Preparation

We extracted 100 μL of plasma using 100 μL of acetonitrile followed by centrifugation for 5 min at 14,000× *g* at 4 °C. This analytical method was established based on the published literature [31,32,33,34]. The supernatant was gathered, evaporated until dry and eventually re-dissolved in 100 μL of 15% acetonitrile. This analytical method was soundly validated in plasma before application.

### 3.4. Liquid Chromatography Coupled to High-Resolution Mass Spectrometry

The detailed parameters for high-resolution mass spectrometry were fully described in our published paper [25]. Briefly, the separation and identification of ZEN-14G and its metabolites were achieved via a gradient elution program on an Acquity HSS T3 column (100 mm × 2.1 mm i.d. 1.7 μm particle size; Waters, Milford, MA) using ACQUITY UPLC (Waters, Co. USA) coupled to a hybrid quadrupole–time of flight mass spectrometer (Q/TOF; SYNAPT HDMS, Waters, UK). The key parameters were source temperature (100 °C), capillary voltage (3.0 kV), cone voltage (35 V), and desolvation gas temperature (300 °C). Low- and high-energy data under collision-induced dissociation were both acquired, with 1.0 ng/μL of leucine encephalin as the internal reference at *m/z* 554.26155. The data acquisition was performed in negative mode.

### 3.5. LC-MS/MS Method and Validation

The separation and identification of ZEN-14G and its metabolites were performed on a Phenomenex column (Kinetex 50 mm × 2.1 mm i.d. 1.7 μm particle size, C18) using an ultra-high-performance liquid chromatograph (Agilent 1260 Infinity) coupled to a triple quadrupole mass spectrometer (Agilent 6495) under negative ESI mode (Agilent Technologies, Palo Alto, CA, USA). The chromatographic separations were achieved through a gradient elution program. Mobile phase A was water containing 0.1% formic acid, and phase B was acetonitrile containing 0.1% formic acid. The gradient elution program for phase B was listed as: 0–1 min, 0%–15%; 1–2 min, 15%–45%; 2–4 min, 45%–90%; 4–5 min, 90%; 5–5.1 min, 90%–15%; and 5.1–6.0 min, 15%. The key parameters for the mass spectrometer were as follows: capillary voltage (3.5 kV); gas temperature (250 °C); gas flow (11 L/min); nebulizer (45 psi); sheath gas temperature (350 °C); and sheath gas flow (12 L/min). Furthermore, the detailed information on precursor ions and the product ions of ZEN-14G and quantifiable products are described in Table S1. This established analytical method was comprehensively validated, where the matrix effects, linearity range, sensitivity, specificity, accuracy, and precision were covered. For method validation, five replicates were conducted at three different spiking concentrations for each compound. For ZEN, α-ZEL, β-ZEL, and β-ZEL-14G, the spiking concentrations were 0.1, 20, and 75 µg/L; for ZEN-14G and α-ZEL-14G, the spiking concentrations were 0.03, 20, and 75 µg/L.

### 3.6. Toxicokinetic Analysis Using WinNonlin 6.3

The obtained data (above LOQ) were analyzed via non-compartmental analysis using WinNonlin 6.3 software (Pharsight Co. Mountain View, CA, USA). The Elimination t_1/2_ can be calculated using the equation t_1/2_ = 0.693/λz, while λz could be obtained from the terminal slope of plotted plasma concentration vs. time curve. The areas under plasma concentration vs. time curve after oral and intravenous administration were for the calculation of absolute bioavailability according to the following equation:

(1) $$F=\frac{\mathrm{AUC}\;\mathrm{oral}\;\mathrm{administration}}{\mathrm{AUC}\;\mathrm{intravenous}\;\mathrm{administration}}\times 100\%.$$

## 4. Conclusions

Generally, the toxicokinetic behavior of ZEN-14G after oral and intravenous administration was specifically investigated in rats in the current study. Our results showed that the oral bioavailability of ZEN-14G was extremely low. The amount of ZEN-14GlcA kept increasing from the first time-point to the last time point after oral administration. Considering the large amount and slow elimination, ZEN-14GlcA is likely to be considered as a biomarker of ZEN-14G. Following intravenous injection, ZEN-14G was rapidly converted into ZEN as a larger amount of ZEN was detected at the first time-point. Moreover, the preparation of standards of ZEN-14GlcA and other major metabolites is the key issue, because the unavailability of standards limited their quantitation in plasma. The acquired data in this study would facilitate the accurate risk assessment of ZEN with regard to exposure to modified mycotoxins.

## Figures and Tables

**Table 1:** The integrated toxicokinetic parameters of ZEN-14G in rats after p.o. and i.v. administration at a dose of 0.75 mg/kg body weight using non-compartmental analysis. The results are shown as mean ± standard deviation (*n* = 12).

Parameters	Unit	p.o.	i.v.
λ_Z_	h^−1^	0.72 ± 0.08	3.18 ± 0.47
Elimination t_1/2_	h	0.96 ± 0.07	0.22 ± 0.03
C_max_	ng·mL^−1^	0.2 ± 0.03	-
t_max_	h	0.083 ± 0.01	-
AUC_last_	h·ng·mL^−1^	0.11 ± 0.03	1.79 ± 0.03
AUC_0-infinity_	h·ng·mL^−1^	0.16 ± 0.02	1.80 ± 0.02
V_Z_	L·kg^−1^	6.24 ± 0.73	7.25 ± 1.23
CL	L·h^−1^·kg^−1^	4.50 ± 0.65	5.02 ± 0.62
MRT	h	0.60 ± 0.05	0.20 ± 0.03
F	%	8.89%	

Note: p.o.: oral administration; i.v.: intravenous administration; λ_Z_: the elimination rate constant; Elimination t_1/2_: the elimination half-life; C_max_: the maximum concentration; t_max_: the time taken to reach the maximum concentration; AUC_last_: the area under the concentration–time curve from 0 to the ultimate blood collection point; AUC_0-infinity_: the area under the concentration–time curve from 0 to the last blood collection time point; V_Z_: the apparent volume of distribution; CL: apparent total body clearance; MRT: mean residence time; F: bioavailability.

## Supplementary Materials

Supplementary materials can be found at https://www.mdpi.com/1422-0067/20/21/5473/s1.

## Abbreviations

ZEN-14G	zearalenone
LC-MS/MS	liquid chromatography tandem mass spectrometry
AUC	area under the plasma concentration–time curve
V_Z_	apparent volume of distribution
CL	total body clearance
DON	deoxynivalenol
LOQ	limit of quantitation
GlcA	glucuronic acid
LC-HRMS	liquid chromatography high-resolution mass spectrometry
