# Supplementary material for: Metabolic Profile, Bioavailability and Toxicokinetics of Zearalenone-14-Glucoside in Rats after Oral and Intravenous Administration by Liquid Chromatography High-Resolution Mass Spectrometry and Tandem Mass Spectrometry
Source: Int J Mol Sci. 2019 Nov 3;20(21):5473. doi: 10.3390/ijms20215473 (PMC6862289; doi:10.3390/ijms20215473)
Supplement: Supplementary file 1 [file ijms-20-05473-s001.pdf]

Supplementary Material

Table S1. Detailed information of ZEN-14G and major metabolites including precursor ions and corresponding product ions, collision energy, linear equation, and sensitivity.

Compound	Precursor Ion (*m*/*z*)	Product Ion (*m*/*z*)	CE (eV)	Elemental Composition	Linearity Range (μg/L)	Regression Equation	r^2	LOD (μg/L)	LOQ (μg/L)
ZEN	317.1 [M-H]-	175.1 [a]<br>131.1	23<br>29	$C_{11}H_7O_3$-<br>C_9H_7O-	0.1–100	Y = 2222.2914X - 107.3044	0.997	0.03	0.1
α-ZEL	319.1 [M-H]-	160.1 [a]<br>188.1	33<br>18	$C_9H_4O_3$-<br>$C_9H_6O_3$-	0.1–100	Y = 1526.5935X - 79.9045	0.999	0.03	0.1
β-ZEL	319.1 [M-H]-	160.1 [a]<br>188.1	33<br>18	$C_9H_4O_3$-<br>$C_9H_6O_3$-	0.1–100	Y = 1721.8353X - 98.3354	0.998	0.03	0.1
ZEN-14G	479.1 [M-H]-	317.1 [a]<br>175.1	16<br>40	$C_{18}H_{21}O_5$-<br>$C_{10}H_7O_3$-	0.03–100	Y = 2736.8353X - 48.8494	0.998	0.01	0.03
α-ZEL-14G	481.2 [M-H]-	319.1 [a]<br>275.1	15<br>34	$C_{18}H_{23}O_5$-<br>$C_{17}H_{23}O_3$-	0.03–100	Y = 3126.4545X - 63.6905	0.992	0.01	0.03
β-ZEL-14G	481.2 [M-H]-	319.1[a]<br>275.1	15<br>34	$C_{18}H_{23}O_5$-<br>$C_{17}H_{23}O_3$-	0.1–100	Y = 2295.981X - 122.075	0.999	0.03	0.1

Where, [a] means quantitation ion; CE stands for collision energy; r^2, correlation coefficient; LOD, limit of detection; LOQ, limit of quantification (S/N > 10), respectively.

Table S2. Integrated results of analytical method validation.

Compound	Low Spiked Level (LOQ)			Medium Spiked Level (5 LOQ)			High Spiked Level (10 LOQ)		
	R_{ave} (%)	RSD Intra-Day (%)	RSD Inter-Day (%)	R_{ave} (%)	RSD Intra-Day (%)	RSD Inter-Day (%)	R_{ave} (%)	RSD Intra-Day (%)	RSD Inter-Day (%)
ZEN	81.2	8.3	9.7	85.6	9.0	10.8	83.9	8.9	10.3
α-ZEL	89.3	9.8	11.3	83.5	8.9	9.7	90.4	9.5	10.6
β-ZEL	92.1	10.6	13.2	88.1	9.6	11.4	86.9	9.2	11.6
ZEN-14G	90.4	12.6	14.8	92.4	13.9	14.9	89.6	12.1	13.5
α-ZEL-14G	89.9	11.2	12.9	90.1	13.2	12.9	85.8	13.8	12.8
β-ZEL-14G	86.5	10.1	11.7	81.7	12.0	10.4	85.7	11.9	13.6

Where R_{ave} represents average recovery; RSD, relative standard deviation.
